# Exploring the Influence of Human–Computer Interaction Experience on Tourist Loyalty in the Context of Smart Tourism: A Case Study of Suzhou Museum

**DOI:** 10.3390/bs15070949

**Published:** 2025-07-14

**Authors:** Ke Xue, Xuanyu Jin, Yifei Li

**Affiliations:** 1 USC-SJTU Institute of Cultural and Creative Industry, Shanghai 200240, China; kxue@sjtu.edu.cn; 2 School of Media & Communication, Shanghai Jiao Tong University, Shanghai 200240, China; jxy0524@sjtu.edu.cn

**Keywords:** human–computer interaction, smart tourism, destination loyalty, cognitive appraisal theory

## Abstract

As digital technology evolves rapidly, smart tourism has become a significant trend in the modernization of the industry, relying on advanced tools like big data and cloud computing to improve travelers’ experiences. Despite the growing use of human–computer interaction in museums, there remains a lack of in-depth academic investigation into its impact on visitors’ behavioral intentions regarding museum engagement. This paper employs Cognitive Appraisal Theory, considers human–computer interaction experience as the independent variable, and introduces destination image and satisfaction as mediators to examine their impact on destination loyalty. Based on a survey of 537 participants, the research shows that human–computer interaction experience has a significant positive impact on destination image, satisfaction, and loyalty. Destination image and satisfaction play a partial and sequential mediating role in this relationship. This paper explores the influence mechanism of human–computer interaction experience on destination loyalty and proposes practical interactive solutions for museums, aiming to offer insights for smart tourism research and practice.

## 1. Introduction

As digital technology progresses rapidly, smart tourism is increasingly becoming a central trend in contemporary tourism development. Smart tourism employs big data, cloud computing, and the IoT to optimize visitor experiences and enhance the operational efficiency of tourism destinations. This transformation has not only altered the operational model of the tourism industry but also compelled various cultural and tourism institutions, particularly museums, to reassess their overall service frameworks and cultural tourism experience designs. In recent years, an increasing number of museums have begun exploring the use of cutting-edge digital technologies and methods to enhance visitors’ viewing experiences and improve their cultural communication effectiveness. Through the innovation of interactive human–computer experiences, museums aspire to increase visitor numbers and reinforce both their cultural significance and social relevance.

While museums are fundamentally committed to cultural education and communication, conventional exhibition formats have become insufficient for addressing the dynamic expectations of visitors in the age of digital media. As tourists increasingly seek more engaging experiences, museums face mounting challenges in adapting to these new demands. Smart tourism has emerged as a result of the deep integration of information technology with the tourism industry, with its core focus on enhancing the travel experience through technological innovation (Chaturvedi et al., 2024). A significant amount of research has explored the technology-driven experience upgrades and has investigated the notable differences between smart tourism experiences and traditional travel experiences in terms of immersion, practicality, usability, and other dimensions (Sustacha et al., 2023). Relevant studies are transitioning from initial explorations of the feasibility of technology applications to more in-depth assessments of user experience quality, value co-creation mechanisms, and the sustainability issues of smart tourism (Chuang, 2023).

The concept of “human–computer interactive experiences”, as a novel service paradigm, enables the application of digital tools to offer visitors diverse and personalized engagements, including interactive exhibits, VR-guided tours, AR technologies, and intelligent navigation systems. These innovations not only cater to the varied needs of different visitors but also inspire deeper interest in cultural understanding and experiences, and they are regarded as having “restorative potential” for enhancing tourists’ psychological well-being (Shen et al., 2024), thereby strengthening visitors’ identification with museums and the cultures that they represent. The focus of human–computer interaction research in museums has transitioned from simply achieving technical functionalities and demonstrating novelty, to a more comprehensive and in-depth assessment of the far-reaching impacts of these technologies on visitors’ cognitive processes, emotional experiences, and behavioral patterns (Lu et al., 2023).

In recent years, studies on tourist loyalty have been on the rise (S. Li, 2024). Highly loyal tourists tend to revisit destinations and promote them via word of mouth, enhancing brand visibility and increasing tourist engagement. Tourist attractions (like smart museums) are an essential part of tourism destinations, and they are a crucial factor in visitors’ satisfaction with the destination while traveling (De Los Reyes & Dael, 2023). The uniqueness of tourist attractions, through attraction loyalty, also significantly affects destination loyalty (Kong et al., 2022). Thus, comprehensively exploring the determinants of destination loyalty, particularly the influence of human–computer interaction in the museum context, has become increasingly critical.

Despite the extensive achievements in existing research on the application of smart tourism technologies, interactive experience design in museums, factors affecting visitors’ loyalty, and the connection between destination satisfaction and loyalty (Gao & Liu, 2024), there remains a gap in systematically addressing how human–computer interaction affects this relationship. The comprehensive effect pathway of human–computer interaction experiences in tourist attractions, on destination image, loyalty, and satisfaction, still has considerable potential for deeper exploration. In the context of museums, a key cultural space, destination loyalty impacts not just revenue generation but also the institution’s broader goals of cultural transmission and education. Understanding how the specific dimensions of human–computer interaction experiences systematically and through multiple pathways affect the mechanisms of destination loyalty formation is essential to explore.

This study takes the Suzhou Museum as a case, aiming to investigate whether the human–computer interaction experience of museums, as destinations, affects visitors’ loyalty to Suzhou as a destination, as well as what roles destination image and satisfaction play within this context. To answer this research question, this study reviews the literature in the field of smart tourism and proposes research variables and their possible interconnections as research hypotheses. Then, employing a questionnaire survey method, this study focuses on visitors to the Suzhou Museum as research subjects, to gather research data and test the hypotheses using structural equation modeling. Ultimately, based on the research findings, strategic suggestions are made for the development of smart tourism, methods to enhance human–computer interaction experiences, and focal points of attention.

This exploration aims to contribute to filling the research gap in smart tourism concerning the relationship between museum human–computer interaction experiences and destination loyalty, and also to provide theoretical support for the construction of smart tourism. Additionally, this study aspires to offer practical and effective suggestions for museums to improve human–computer interaction experiences and boost visitors’ destination loyalty, supporting their sustainable development and, thus, facilitating the deep integration of culture and tourism.

## 2. Literature Review

### 2.1. Research on Smart Tourism

In recent years, the global tourism sector has encountered unprecedented challenges, including the COVID-19 pandemic and geopolitical conflicts. Against this backdrop, the concepts of “Recovery Strategies” (Yeh, 2021) and “Tourism Transformations” (Pung et al., 2020) in the tourism sector have emerged as shared focal points of interest in both academia and industry. Traditional recovery strategies typically emphasize marketing and short-term incentives, whereas more proactive research highlights the need to create more resilient and sustainable tourism systems (Bulchand-Gidumal, 2022). In this context, smart tourism has been introduced as a strategy for tourism transformation and has evolved into a significant area of study (Y. Li et al., 2017).

Smart tourism is a new form of development based on cutting-edge information and communications technology (ICT), which refers to the burgeoning phenomenon in which tourism destinations, practitioners, and tourists depend accumulatively on emerging ICT that enables colossal data transformation into value propositions (Gretzel et al., 2015). At present, smart tourism has entered its fourth stage—intelligentization (Cai et al., 2023), integrating emerging technologies such as 5G, big data, artificial intelligence, and large models into travel experiences to create more systematic, seamless, comfortable, and personalized journeys. Existing studies regard smart tourism as an advanced stage of tourism informatization that focuses on digital tourist experiences (Y. Li et al., 2014) to effectively meet emerging demands in tourism development (L. Zhang et al., 2012). The implementation of smart tourism largely depends on enabling technologies like blockchain (Ming & Wei, 2021), augmented reality (Z. Chen & Wang, 2020), and big data (Semanjski, 2015) to support the development of intelligent destinations and smart cities.

As a crucial part of smart tourism, “attractions” are defined as specific places or entities that attract visitors for sightseeing, exploration, and experiences. Gunn (2014) defines attractions as “specific locations or entities in the tourism system that have the capacity to attract visitors.” Lew (1987) highlights that attractions are central to tourism activities and serve as one of the main motivations for visitors. Attractions can be classified according to their nature into natural attractions (such as national parks), cultural attractions (such as museums), and artificial attractions (such as amusement parks). Their defining feature is a unique attractiveness that can engage visitors’ interest and encourage tourism behavior (Ngwira & Kankhuni, 2018). Attractions serve as the fundamental unit of tourism supply, and they are the direct medium for visitor experiences. Meanwhile, “destination” is a broader and more integrated concept. Buhalis (2000, p. 97) defines a destination as “a geographic area, such as a country, region, city, or specific resort, which is viewed by visitors as a singular entity and is composed of a variety of tourism products and services, which together deliver a comprehensive tourism experience”.

### 2.2. Research on Digital Heritage and Human–Computer Interaction

With the rapid development of digital technology, the preservation, interpretation, and dissemination of digital heritage has emerged as a crucial topic in the cultural field. Digital heritage refers to cultural heritage resources that exist in a digital format or are preserved and displayed through digitization. This includes the digitization of tangible cultural heritage, the digital representation of intangible cultural heritage, and native digital cultural content (Cipolla Ficarra, 2010). Digital heritage is invaluable for cultural transmission, academic research, educational dissemination, and public participation, and it has become a significant component of cultural tourism. For cultural tourism, museums have emerged as significant attractions for many visitors. With the aid of digital technologies, particularly VR and AR, the interactivity and immersive experience of cultural heritage have improved, enhancing museums’ reach and public participation (L. Wang, 2022), and leading to the emergence of a new form: the “digital cultural museum”.

A key strength of the digital cultural museum lies in its ability to transcend the time and space constraints inherent to traditional museum settings. Using digital tools like immersive naked-eye 3D, audiences are now able to view cultural relics and historical locations at any time and from any place through online platforms. Such virtual visits offer tourists innovative forms of exhibition and interaction (J. Wang et al., 2023), while enabling the long-term conservation of heritage artifacts. Additionally, Weng and colleagues suggest that VR technology facilitates a deeper emotional bond between tourists and cultural heritage by offering immersive experiences (J. Zhang et al., 2024). This view is echoed by Theodoropoulos and Antoniou (2022), who similarly argue that VR and AR technologies greatly enhance visitors’ interactivity and involvement, ultimately boosting satisfaction and loyalty. Among all studies related to digital cultural museums, the core concept frequently referenced is “Human–Computer Interaction” (HCI), a field that explores the mutual influence between people and computing systems (Sun & Sun, 2013), including multimodal interfaces such as gaze tracking, gesture and sign language recognition, and speech synthesis (Dong, 2004). The aim of HCI is to improve the accessibility, usability, and user experience of digital heritage (Hirsch et al., 2024). With well-designed HCI, complex cultural information can be conveyed more efficiently in museums, and abstract historical concepts can be represented more vividly (Cesário & Campos, 2024).

In conjunction with past studies on measuring human–computer interaction experiences, user experience in human–computer interaction can be generally categorized into four key elements: immersion, personalization, responsiveness, and controllability (Badu-Baiden et al., 2022). “Immersion” is a central concept in areas such as HCI and smart tourism. It denotes a psychological state in which users become deeply engaged, highly focused, and even temporarily forget the real world during their interactions with media or environments (Robaina-Calderín et al., 2023). Studies on immersion within HCI mainly examine measurement techniques, influencing factors, and their impact on user behaviors and attitudes (Evans & Rzeszewski, 2020). The findings show that a high degree of immersion can significantly improve users’ enjoyment, memory retention, and brand perceptions, as well as enhancing their behavioral intentions. For example, in museum contexts, immersive human–computer interaction experiences can boost visitors’ satisfaction and likelihood of returning (Liu & Sutunyarak, 2024). On the internet, “Personalization” refers to the ability of developers to analyze user behavior data to identify preferences and provide targeted services (Hewei, 2022). Perceived personalization highlights whether the services that users experience can fulfill their individual needs. Well-designed personalized recommendations can reduce the search time for users during product use (Zanker et al., 2010), while highly personalized advertisements can lead to more positive brand engagement from users, thereby enhancing brand loyalty (C. Li, 2016). “Responsiveness” indicates the system’s ability to swiftly respond to user instructions and deliver services or products (Srinivasan et al., 2002), and positive responses can greatly increase users’ trust in the platform (Shanahan et al., 2019). Furthermore, the ability to provide instant and convenient travel service bookings via digital technology platforms is a crucial element of smart tourism. A significant reason why users appreciate the internet is that it enables them to control the content and timing of their interactions (Lee, 2005), and “Controllability” gauges whether users can freely choose the content, sequence, and timing that they wish to view in the human–computer interaction experience.

### 2.3. Research on Destination Tourism

Destination image is regarded as a key variable in research on urban areas and attractions (Berthon et al., 1996). Crompton (1979) defined destination image as the trust, knowledge, and impression that a tourist holds toward a specific destination, which serves to assess one’s subjective perceptions and beliefs about the place (P. J. Chen et al., 2013). The concept of destination image generally includes two main elements: cognitive and affective components (Crompton, 1979). Cognitive image relates to tourists’ perceptions of a destination’s functional attributes, whereas affective image pertains to their emotional responses and psychological experiences (Baloglu & McCleary, 1999). Nonetheless, in research focused on urban imagery and similar fields, the use of cognitive city image alone is also considered valid (Gartner, 1993). Tourists’ destination image is often shaped and altered by their onsite travel experiences (D. Li et al., 2022), and high-quality tourism experiences are a key condition for forming positive cognitive evaluations of a destination (Palau-Saumell et al., 2016). As previously stated, attractions, such as smart museums, serve as crucial parts of destinations. The loyalty to these attractions also significantly influences destination loyalty (Kong et al., 2022). Studies investigating how specific experiences at attractions (like exhibition interactivity and service quality) influence visitor satisfaction, and how these factors further translate into tourism intentions towards the city where the attraction is located (including revisits, recommendations, donations, etc.), have also been a major focus of research (Leo et al., 2021). Accordingly, this study puts forward the following research hypothesis:

**H1.**  *Human–computer interaction experience significantly and positively influences tourists’ perceptions of the destination image.*

Satisfaction represents a positive emotional response that arises during an individual’s experiential process (Tsaur et al., 2017). Visitors’ satisfaction is derived from the comparison between their expectations prior to the trip and the experiences that they actually encounter during the journey (Zhou et al., 2023). Interactive smart technologies in museums prompt tourists to engage more actively, thereby deepening their comprehension of cultural artifacts and simultaneously increasing their overall satisfaction with the museum experience (Ozturk & Gogtas, 2016). Positive experiences in museums, as an element of the city tourism experience, can indirectly contribute to tourists’ overall satisfaction with the destination (Tsaur et al., 2017). A positive destination image stems from favorable tourist evaluations of their destination experience (Dai & Chen, 2019), which often leads to more positive emotional states (Chi & Qu, 2008), thereby enhancing satisfaction. A significant body of research supports the positive impact of destination image on visitor satisfaction. Baloglu and McCleary (1999), in their study of Taiwanese leisure farms, identified a significant positive influence of destination image on tourists’ satisfaction with the destination. Accordingly, this study proposes the following hypotheses:

**H2.**  *Human–computer interaction experience significantly and positively influences destination satisfaction.*

**H3.**  *Destination image significantly and positively influences destination satisfaction.*

The concept of loyalty was originally employed by economists to examine customer preferences toward particular products, services, or brands. With the deepening of academic inquiry, loyalty was incorporated into tourism studies, where destination experiences were conceptualized as products to explore tourists’ revisit intentions and behaviors. A superior tourism experience can significantly increase tourists’ intention to revisit a destination (H. Zhang et al., 2018). Prior research has revealed that tourists’ perceived experience quality, including infrastructure and local characteristics, has a positive influence on their loyalty toward the destination (W. Wu & Tang, 2022). As museums play a key role in a city’s cultural identity, enhanced museum experiences often strengthen visitors’ appreciation of local culture, thereby motivating them to return and recommend both the city and the museum. Satisfied tourists are more inclined to return and positively promote the destination to others (Bao & Hu, 2008), which are critical measures of destination loyalty. The influence of destination satisfaction on loyalty has been widely validated in previous studies. Tourists’ favorable perceptions of destination image are associated with stronger behavioral intentions, including revisits and recommendations (Prayag, 2009). Therefore, the effect of destination image on tourist loyalty demonstrates a certain level of generalizability. In light of this, the following research hypotheses are proposed:

**H4.**  *Human–computer interaction experience exerts a significant positive influence on tourists’ loyalty to the destination.*

**H5.**  *Destination satisfaction significantly and positively influences tourist loyalty.*

**H6.**  *Destination image has a significant positive impact on tourists’ loyalty to the destination.*

### 2.4. Cognitive Appraisal Theory

Cognitive Appraisal Theory (CAT) was first proposed by Richard in 1984 to explain changes in human responses when faced with external stimuli (Ma et al., 2013). The theory posits that individuals’ evaluations of external stimuli occur in two stages: the primary appraisal assesses the level of threat that the stimulus poses to the individual, and if it is perceived as inconsistent with personal goals, a secondary appraisal is triggered to evaluate available resources and coping options, ultimately leading to the adoption of the most appropriate response strategy (Lazarus & Folkman, 1984). Prior research has confirmed the applicability of CAT in multiple disciplines, such as psychology, marketing, and tourism (Chang et al., 2022). Drawing on CAT and script theory, Manthiou and colleagues constructed a multidimensional model of cruise travelers’ repurchase behavior, using the cruise experience as the stimulus and integrating emotional responses, memory, and storytelling (Manthiou et al., 2017).

Current research on smart cultural tourism seldom investigates the psychological mechanisms shaping tourists’ behavioral intentions, a gap that CAT is well positioned to address. This study conceptualizes the human–computer interaction experience perceived by tourists during museum visits as the initial stimulus in the model. Based on this experience, tourists conduct an initial cognitive appraisal (destination image), which then elicits an emotional response (satisfaction). Subsequently, through both cognitive and emotional evaluations, tourists develop intentions to revisit or recommend the destination—referred to as destination loyalty. Accordingly, the following hypotheses are proposed:

**H7.**  *Destination image serves as a mediator in the effect of human–computer interaction experience on destination loyalty.*

**H8.**  *Destination satisfaction functions as a mediator in the relationship between destination image and destination loyalty.*

**H9.**  *Destination image and destination satisfaction jointly serve as serial mediators in the effect of human–computer interaction experience on destination loyalty.*

## 3. Methodology

### 3.1. Research Objectives

**Figure 1:**
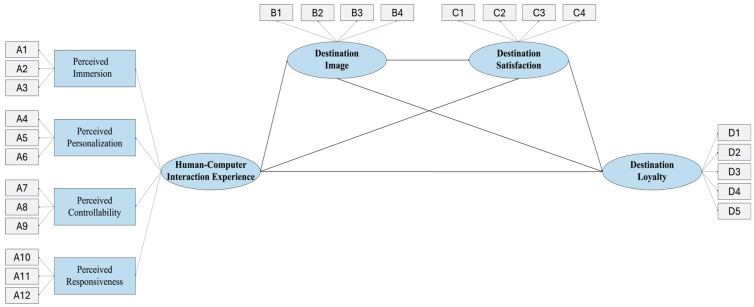
Research model.

Based on the research hypotheses proposed in the literature review, this study establishes a research model (Figure 1). This study is dedicated to examining the interplay between human–computer interaction experience and tourist destination loyalty using this model, while exploring the strength of the mediating effects of destination image and satisfaction.

This study takes the Suzhou Museum as a research case, due to its status as a cultural symbolic landmark of Suzhou and a key destination for numerous tourists, featuring a variety of interactive exhibition technologies and experiences such as VR/AR, digital performances, and gaming elements. Culturally, Suzhou Museum seamlessly blends historical artifacts with traditional Suzhou culture, incorporating modern structures, ancient architecture, and classical landscapes to showcase the distinctive allure of Gusu City (Q. Zhang, 2019). Technologically, Suzhou Museum is one of the initial pilot units for smart museum development in China, with intelligent systems, visual technologies, and interactive tools embedded in both its exhibition design and museum operations. Through these interactive elements, tourists can immerse themselves in Suzhou’s urban culture and folk traditions. Therefore, visitors to Suzhou Museum in the past three months were selected as the target sample for this study.

Regarding the measurement of research variables, this study is based on the work of Badu-Baiden et al. (2022) and evaluates the human–computer interaction experience variable across four dimensions: “Perceived Immersion,” “Perceived Personalization,” “Perceived Controllability,” and “Perceived Responsiveness.” The “Perceived Immersion” dimension is derived from the scale developed by Jennett et al. (2008), consisting of three items, including “I find the interaction process of the interactive devices at the Suzhou Museum very natural” (A1–A3). The “Perceived Personalization” dimension draws from the scale by Luan et al. (2023), comprising three items, including “The interactive devices at the Suzhou Museum can offer me a variety of services” (A4–A6). The dimensions of “Perceived Controllability” and “Perceived Responsiveness” are based on the scale by Yoo et al. (2010), consisting of three items each, such as “The interactive devices at the Suzhou Museum are very user-friendly” and “I can quickly obtain information from the interactive devices at the Suzhou Museum” (A7–A12). The “Destination Image” dimension is derived from the scale developed by Chaulagain et al. (2019), consisting of four items, including “The Suzhou Museum has a well-established tourism information network” (B1–B4). The “Destination Satisfaction” dimension draws from the scale developed by Bigné et al. (2005), comprising four items, including “I am very happy to have visited the Suzhou Museum” (C1–C4). The “Destination Loyalty” dimension references the scale developed by Yu and Hwang (2019), including five items, such as “I intend to revisit the Suzhou Museum” (D1–D5).

### 3.2. Pilot Study

The questionnaire used in this study was constructed by referencing established scales and adjusting them in accordance with the research hypotheses. Upon completion of the questionnaire design, a preliminary survey was launched using the Wenjuanxing platform to verify the reliability and validity of the items, thus guaranteeing the effectiveness of the formal study. The pilot study obtained 95 responses, with 89 deemed valid, resulting in a valid response rate of 93.68%. SPSS 26.0 was employed for data analysis, with the KMO value reported at 0.801, confirming the adequacy of the data for validity testing. Bartlett’s test of sphericity produced a chi-squared value of 1594.400 (df = 378, *p* < 0.05), indicating that the data met the criteria for statistical significance. Thus, the dataset satisfies the validity requirements and is suitable for subsequent factor analysis. A principal component analysis was performed, and the rotated component matrix was obtained (Table 1). The results showed that all of the item loadings were above 0.7, and the grouping of items aligned with the theoretical constructs, providing additional support for the validity of the instrument, and indicating no need for modifications.

### 3.3. Research Design

To empirically test the research hypotheses, this study conducted a formal questionnaire-based survey between 3 July and 17 July 2024, at the Suzhou Museum. The survey instrument was developed based on the prior pilot study, and both its reliability and validity were rigorously assessed and confirmed during the pretest phase.

Data collection was carried out onsite by trained members of the research team using an online survey format (hosted via a secure platform), which the respondents accessed by scanning a QR code. The survey was administered in the main lobby of the Suzhou Museum during three designated time slots per day—morning, noon, and late afternoon—each lasting approximately two hours, to ensure temporal diversity and reduce potential time-based sampling biases.

The participants were approached randomly as they entered or exited the lobby area. Upon initial contact, a research team member briefly introduced the purpose of the study, estimated time commitment, and the anonymous nature of participation. Informed consent was obtained both verbally and digitally. Specifically, the participants were first asked for verbal agreement to participate, after which they were invited to scan the QR code leading to the online questionnaire. At the beginning of the questionnaire, a digital informed consent form was presented. The participants were required to read and confirm their agreement before gaining access to the main body of the survey. The consent form clearly stated that participation was entirely voluntary, the responses would remain anonymous, and the data would be used solely for academic purposes.

### 3.4. Sample Description

SPSS 26.0 was employed to perform descriptive statistical analysis on gender, age, educational background, and the extent to which the respondents perceived Suzhou’s culture during their visit to Suzhou Museum, in order to understand the distribution of the data (Table 2). The gender distribution was relatively even, with 293 males (54.6%) and 244 females (45.4%) participating in the study. The participants were primarily in the 18–30 age group, which accounted for 44% of all respondents. In terms of education, the sample included participants with junior high school or below (20.7%), high school/secondary vocational education (25%), and associate college degrees (27%). Approximately 60.5% of the participants indicated that they experienced Suzhou’s culture during their museum visit, suggesting that Suzhou Museum serves as a strong cultural representative, and that the sample selection is appropriate and representative.

## 4. Research Findings

### 4.1. Model Validity Test

**Figure 2:**
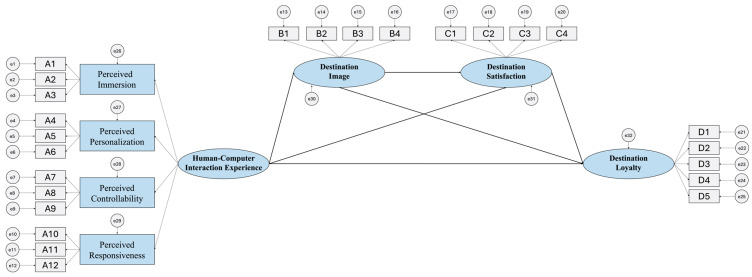
Structural equation model.

The validity of the model was assessed primarily through confirmatory factor analysis (CFA). The evaluation was based on χ^2^/df (threshold < 3), RMSEA (threshold < 0.08), and other fit indices, including the GFI, AGFI, CFI, and IFI, all of which should exceed 0.90. Additionally, convergent validity and discriminant validity were applied to evaluate the construct validity of the questionnaire. Convergent validity is deemed satisfactory when the AVE is above 0.5, CR exceeds 0.7, and standardized factor loadings surpass 0.6. In accordance with the theoretical model and hypotheses, AMOS 24.0 was used to develop a structural equation model incorporating human–computer interaction experience (perceived immersion, personalization, controllability, and responsiveness), destination image, satisfaction, and loyalty. The model included a total of 25 observed variables (e1~e25) and 7 latent variables (e26~e32). Six hypothesized paths were established based on the theoretical framework, and their validity was tested through path coefficients and significance levels. The structural equation model is presented in Figure 2.

Model validity was assessed through confirmatory factor analysis (CFA), using commonly accepted fit indices such as the chi-squared (χ^2^) test and its ratio to degrees of freedom (χ^2^/df), where a value less than 3 typically indicates a good model fit. The root-mean-square error of approximation (RMSEA) was also examined, with values below 0.08 indicating a high degree of model fit. The goodness-of-fit index (GFI) and adjusted goodness-of-fit index (AGFI) are generally considered acceptable when above 0.8, while the comparative fit index (CFI) and incremental fit index (IFI) should exceed 0.9, indicating a good model fit. The CFA model fit indices are summarized in Table 3. The results confirm that the structural equation model demonstrates a satisfactory level of fit according to established academic standards.

### 4.2. Direct Path

Based on the structural model diagram, this study examined the effects among variables through path coefficients and their significance levels, with the model validation results presented in Table 4. The path analysis revealed that the effect sizes, both raw and standardized, between the paired variables were statistically significant. These findings confirm the validity of hypotheses H1 through H6.

### 4.3. Intermediary Effect

In order to investigate the mediating roles of destination image and destination satisfaction, this research utilized the bootstrapping method in AMOS 24.0, running 5000 iterations. The significance of the mediation effects was evaluated under a 95% confidence interval. If the bootstrap confidence interval excluded zero, the corresponding path effect was considered significant. Table 5 presents the summary of the mediation effect test results. In the examination of the three mediation paths in the table, the significance of the total effect was determined based on the total and indirect effect values. The research data indicate that, after accounting for destination image, the direct effect of human–computer interaction experience on destination loyalty was still significant. Similarly, controlling for destination satisfaction still resulted in a significant direct effect of human–computer interaction experience on destination loyalty. Even when both destination image and satisfaction were controlled, the direct effect of human–computer interaction experience on destination loyalty persisted significantly. This indicates that all three paths exhibit partial mediation effects.

## 5. Research Conclusions

This study adopts a user-centric perspective and situates destination research within the context of emerging technologies. It seeks to examine the impact of smart technologies on tourists’ visiting experiences, cognitive perceptions, and willingness to revisit. The study collected respondents’ data using a questionnaire-based survey approach. It aimed to test the hypothesis that tourists’ human–computer interaction experiences in museums influence their perception of the destination image. The museum, serving as a highly condensed representation of urban culture, was used as the primary research setting. This approach aimed to enhance conceptual clarity regarding “human–computer interaction experience” among respondents, and to ensure model validity. The results showed that human–computer interaction experience significantly and positively affects destination image, satisfaction, and loyalty. Additionally, the interrelations among the three were empirically tested. This study extends the applicability of Cognitive Appraisal Theory in destination research. Furthermore, it offers novel theoretical insights for the advancement of smart tourism in cities.

### 5.1. HCI Experience Positively Affects Destination Loyalty

The study found that HCI experiences significantly and positively enhance tourists’ intentions to revisit and recommend the destination—an effect that had not been empirically validated in previous research. However, a high-quality interactive experience can indeed create an environment that encourages tourist engagement, thereby enhancing their perceived value of the visit (Zheng et al., 2024). The perception of value is a crucial determinant in fostering tourists’ behavioral intentions (J. Wu et al., 2022). The findings verify that an effective HCI experience serves as the foundation for boosting destination loyalty, filling the empirical void between these variables. Among the four dimensions of tourists’ HCI experience, perceived control over the device exerts the greatest influence. This is followed by personalization and immersion, while responsiveness has the least effect. Rather than response speed, users place greater emphasis on whether they can control the content and sequence of information access. Individuals do not wish to feel dominated by smart technologies (Fast & Schroeder, 2020). Hence, during interactions with smart devices, tourists who perceive autonomy in choosing their interaction content and methods are more likely to experience autonomy, encouraging ongoing engagement (Y. Huang et al., 2016). The response speed of smart technologies plays a role in shaping tourists’ interaction experiences within museums. Despite a lack of direct empirical evidence, prior studies consistently affirm that perceived responsiveness positively influences users’ behavioral intentions. Steuer (1992) highlighted that the ability to modify media’s content and form in real time is a central aspect of interactivity.

### 5.2. HCI Experience Positively Affects Tourists’ Destination Cognition and Emotion

Our findings reveal that HCI experiences exert a notable positive impact on tourists’ cognitive understanding and emotional perception of destination image. In particular, the use of HCI technologies leads to more favorable cognitive and emotional responses toward the destination among tourists. Prior research confirms that AR and image recognition technologies similarly enhance tourists’ destination perception and emotional attachment, reinforcing their identification with the locale (H. Zhang & Liao, 2024). HCI technologies have also proven essential in enhancing user experiences within touchless tourism services. Taking an island fishing village as a case in point, tourists are able to utilize interactive systems for self-service, thus bypassing complex manual operations. Such services are efficient and human-centered, improving tourists’ satisfaction while also fostering a stronger identification with the destination. Similarly, the smart interactive design of sightseeing trains enables tourists to access real-time destination information and experience virtual cultural elements via smart screens inside the cabins (R. Wang & Liu, 2024). HCI experiences have a significant positive effect on enhancing tourists’ destination image and emotional engagement. From immersive VR and real-time AR interaction to contactless self-services, all of these technologies enrich the cultural experience and offer greater convenience for tourists. This not only enhances tourists’ satisfaction but also reinforces their emotional ties to the destination, which deepens their loyalty and encourages recommendation behaviors. This result offers theoretical backing for the advancement of smart tourism and underscores the pivotal role of HCI in forming destination images and enriching emotional tourist experiences.

### 5.3. Path of Influence of HCI Experience on Destination Loyalty

Grounded in Cognitive Appraisal Theory, this study verifies that HCI experiences influence destination loyalty by first enhancing tourists’ perceptions of destination image, which subsequently boosts their satisfaction. While numerous prior studies have examined the correlations among destination image, satisfaction, and loyalty, most tend to isolate the relationship between just two of them. There is a lack of research models connecting HCI experience simultaneously to destination image, satisfaction, and loyalty. The current model fills this gap by offering a psychological pathway from HCI experience to tourist loyalty toward a destination. Earlier research typically addressed the mediating role of destination image or satisfaction individually. The mediating effects of both factors were assessed here, revealing that both image and satisfaction significantly mediate the impact of HCI on loyalty, with satisfaction (c = 0.310) playing a slightly more prominent role than destination image (c = 0.153). This reflects that during interactions with smart technologies, emotional activation tends to have a stronger effect on behavioral intentions than cognitive enhancement. Ultimately, the model confirms that HCI experience indirectly and significantly influences tourist loyalty through destination image and satisfaction. C.-C. Huang and Lin (2023) also validated this sequential mediation effect in their research on Taiwan’s Gaomei Wetlands, where they observed that destination image and satisfaction jointly mediate the impact of travel learning on destination attachment. Relative to the traditional SOR model, using Cognitive Appraisal Theory offers a better framework for understanding how HCI influences behavioral intentions, since emotions significantly shape how individuals respond to external stimuli (Faiola et al., 2013).

## 6. Research Discussion

### 6.1. Smart Museums Help Urban Cultural Brand Building

With the rapid development of information technology, museums serve as essential platforms for cultural communication and play an irreplaceable role in smart cultural heritage construction. The smart museum is not only a medium for presenting artifacts and history but also a vital channel for cultural preservation and innovation. Thus, museums must prioritize improving the quality of human–computer interaction by incorporating technologies such as AR, VR, and smart devices to deliver layered cultural content and create multidimensional immersive experiences that foster visitors’ cultural identification. Museums should also work on crafting a cohesive public image that is capable of emotionally connecting with visitors. For instance, by developing culturally themed interactive content reflecting local traits, museums can strengthen visitors’ cultural identification. When museums deeply present their cultural uniqueness, visitors experience the depth of their historical and cultural value, which inspires affection and encourages increased loyalty.

The smart museum serves as a significant attraction in urban development and is a key cultural institution, with its long-term planning needing to fully incorporate cultural traits and audience demands. Cultural institutions, as stewards of cultural resources, can digitize cultural resources like collections and exhibitions, creating virtual exhibitions, cultural apps, and other offerings that can enrich the cultural depth and experiential aspects of tourist sites, ultimately enhancing the cultural added value and appeal of tourism products. As living standards rise and cultural consumption habits evolve, tourists are demanding higher quality in their travel experiences, with a stronger focus on participation, interactivity, and personalization. Developing tourism products guided by audience needs can boost visitor satisfaction and loyalty (Ramesh & Jaunky, 2021). Smart museums and similar attractions can utilize big data analytics and AI technologies to gain a deeper understanding of audience cultural interests and preferences. This enables accurate market positioning and product design recommendations for attractions. For instance, by analyzing visitors’ browsing records, search keywords, and other data on the smart museum platform, along with interaction durations and frequencies across various devices in the museum, museums can gauge visitors’ interest in specific historical and cultural themes. Based on these insights, attractions can create related cultural theme activities and customized travel itineraries, catering to the diverse needs of various visitor demographics and, thus, achieving a sustainable development model for smart tourism.

In addition, building smart cultural museums has long-term strategic implications for enhancing urban smart tourism. Museums, as pivotal components in the smart tourism ecosystem, can synergize with other smart tourism initiatives to strengthen the cultural attractiveness of a city. Resource sharing and data integration with other smart attractions can enable smoother transitions for tourists across different sites and offer them a broader spectrum of cultural services. For example, museums may collaborate with other cultural venues to issue integrated smart tickets or build multi-location smart guide systems to simplify itinerary planning for visitors. A holistic and diversified smart tourism system not only improves travel efficiency and enjoyment but also reinforces the city’s cultural branding and enhances its soft cultural power.

### 6.2. Museums Should Enhance HCI Experiences in Multiple Dimensions

This study demonstrates that HCI experiences significantly enhance tourists’ loyalty. This implies that introducing immersive intelligent interaction experiences in cultural tourism venues such as museums can effectively stimulate visitors’ intention to revisit. Meanwhile, such interactive experiences also stimulate tourists’ willingness to recommend the destination to others. This results in strong word-of-mouth marketing among visitors and reflects a high level of destination loyalty. It lays a solid groundwork for the sustainable development of cultural attractions. This study identified four core experiential factors: immersion, personalization, responsiveness, and a sense of control. Each of these factors contributes positively to tourist loyalty. Among them, perceived controllability exerts the most significant influence on tourist loyalty. This indicates that when tourists are able to independently select and control content during interactive experiences, their sense of identification and belonging with the museum is strengthened, thereby enhancing their loyalty. This underscores the key role of perceived autonomy in influencing visitors’ behavior. It offers valuable insights for museums and similar institutions in crafting effective interactive technologies.

Additionally, the distinctive and significant role of Destination Management Companies (DMCs) and Destination Marketing Organizations (DMOs) in improving human–computer interaction experiences should not be ignored. DMCs can integrate the technological resources and service providers of the destination, bringing in advanced and appropriate technological solutions for museums, such as immersive virtual exhibitions and interactive guide applications, thus offering visitors a more enriched and personalized experience. DMOs emphasize creating the overall brand image of the destination from a marketing perspective, driving the improvement of human–computer interaction experiences in museums, to strengthen the cultural appeal and competitiveness of the destination. Furthermore, DMOs can integrate the human–computer interaction experiences of museums into the overall tourism promotion strategies of the destination, by publicizing and promoting these innovative interactive experience initiatives, drawing more tourists to visit, thus increasing the cultural tourism allure of the destination. Only through actively combining the various resources of the tourism destination (Sangpikul, 2018) and mobilizing the participation of diverse local stakeholders can we assist museums in creating a more appealing and interactive visiting atmosphere, thus driving the high-quality development of cultural tourism in the destination.

### 6.3. Positive Emotional Arousal Is the Core Goal of Offline Experience

Destination image and visitor satisfaction play a critical mediating role between visitor experience and loyalty. Tourists’ positive cognitive and emotional evaluations of the museum are not solely dependent on the quality of intelligent interaction. They are also deeply affected by the overall image of the museum. Specifically, high-quality human–computer interaction provides visitors with a strong sense of immersion and enjoyment. This facilitates the formation of positive emotional bonds during their visit. As these positive emotions accumulate, visitors develop a stronger sense of identification and belonging toward the museum. Such emotional attachment further motivates their revisit and recommendation behaviors. For example, when tourists gain deeper insights into the museum’s history, culture, and artistic value through interactive experiences, their sense of knowledge acquisition and emotional resonance gradually increases. This, in turn, enhances their emotional identification with the museum. Consequently, this sense of identification translates into more positive behavioral intentions, making them more likely to revisit the museum and recommend it to friends or family in the future.

In light of the research findings, destination image and satisfaction serve as chain mediators in the relationship between interactive experience and loyalty. It can be observed that emotions are the final focal point of the museum’s offline interactive experience as perceived by visitors. Human–computer interaction experiences can draw visitors into specific themes within the museum, while a more holistic cultural experience can deliver strong sensory enjoyment to visitors in a brief period, leading to strong positive emotional responses and fostering more enduring and profound memories of their experience. This finding is corroborated by the research conducted by Deng et al. (2024); thus, when visitors recall this experience, these memories can trigger strong emotional resonance in visitors and encourage them to form more positive behavioral intentions.

Therefore, when designing and implementing human–computer interaction experiences, museums should take into account both tourists’ cognitive needs and emotional experiences. Through content innovation and technological application, dual stimulation of cognition and emotion can be achieved. Maximizing the enhancement of the visitor experience can not only help foster stronger psychological bonds among visitors but also attract more potential tourists through word of mouth, thus laying a solid foundation for the sustainable development of museum tourism.

## 7. Research Limitations and Prospects

This study acknowledges several limitations in examining the impact of human–computer interaction experience on visitors’ destination loyalty in the context of the Suzhou Museum. First, the data used in this study were primarily derived from visitors to the Suzhou Museum, leading to a distinct regional limitation in the sample. This may restrict the generalizability of the findings to other museums or cultural tourism sites. Therefore, future studies should consider collecting data from a variety of cultural institutions, in order to enhance the external validity and generalizability of the research findings. Second, participants’ personal interests, social groups, life experiences, and family background, as well as their familiarity with digital technologies like VR/AR, may also affect their perceptions of human–computer interaction at attractions, thereby influencing their loyalty to the destination. These demographic variables, as well as familiarity with technology, could serve as moderating variables to be tested in future studies to investigate their influence. Third, this research is based on cross-sectional data, limiting the capacity to explore the dynamic evolution of destination loyalty. This, in turn, reduces the ability to anticipate long-term patterns in visitor behavior. Therefore, future research should adopt a longitudinal research design to gain a better understanding of the temporal evolution of destination loyalty and to capture the dynamic processes underlying such behavior.

## Figures and Tables

**Table 1:** Composition matrix after rotation.

	Components						
	1	2	3	4	5	6	7
A1	0.076	−0.007	0.045	**0.874**	0.096	0.159	0.094
A2	0.064	0.026	0.134	**0.855**	0.068	0.088	0.204
A3	0.158	−0.088	0.168	**0.702**	0.182	0.277	0.072
A4	0.122	0.253	−0.040	0.270	**0.767**	0.103	−0.068
A5	0.134	0.004	0.119	0.086	**0.848**	0.147	0.172
A6	0.167	0.023	0.167	0.056	**0.854**	0.153	0.165
A7	0.186	0.129	0.014	0.173	0.053	0.043	**0.813**
A8	0.065	0.031	−0.122	0.208	0.054	0.088	**0.821**
A9	0.173	0.146	0.132	−0.017	0.171	0.163	**0.839**
A10	0.079	0.166	0.190	0.136	0.103	**0.826**	0.119
A11	0.118	0.036	0.185	0.187	0.103	**0.801**	0.092
A12	0.017	0.155	0.075	0.217	0.222	**0.771**	0.108
B1	0.241	0.265	**0.697**	0.097	0.035	0.193	−0.018
B2	0.275	0.194	**0.727**	0.187	0.089	0.109	0.048
B3	0.218	0.160	**0.675**	0.222	0.022	0.232	−0.087
B4	0.132	0.161	**0.874**	−0.005	0.147	0.092	0.043
C1	0.264	**0.823**	0.187	−0.033	0.011	0.079	0.216
C2	0.197	**0.788**	0.223	0.081	−0.003	0.090	−0.044
C3	0.270	**0.812**	0.224	0.019	0.112	0.031	0.046
C4	0.192	**0.641**	0.050	−0.201	0.201	0.256	0.267
D1	**0.771**	0.147	0.173	0.140	0.145	0.075	0.054
D2	**0.70**	0.206	0.240	0.088	0.116	0.060	0.091
D3	**0.752**	0.203	0.128	−0.063	0.172	0.087	0.146
D4	**0.793**	0.262	0.099	0.129	−0.005	−0.026	0.123
D5	**0.839**	0.124	0.164	0.065	0.091	0.109	0.127

The data in bold signify the absolute value of load factors greater than 0.6, suggesting that these factors can be categorized under the same variable.

**Table 2:** Analysis of the basic situation of the respondents.

Variable	Value	Frequency	Percentage
Gender	Male	293	54.6
	Female	244	45.4
Age	Under 18 years old	66	12.3
	18~25 years old	111	20.7
	26~30 years old	125	23.3
	31~40 years old	76	14.2
	41~50 years old	83	15.5
	51~60 years old	43	8.0
	Over 60 years old	33	6.1
Academic degree	Junior high school and below	111	20.7
	High school/technical secondary school	134	25.0
	Junior college	145	27.0
	Undergraduate college	103	19.2
	Graduate students and above	44	8.2
I felt the culture of Suzhou in Suzhou Museum.	Yes	325	60.5
	No	212	39.5

**Table 3:** Model fit index analysis.

Fitting Index		Evaluation Standard	Result	Model Fitting Judgment
Absolute fitting index	χ^2^	/	510.256	/
	*df*	/	322	/
	χ^2^/df	<3	1.585	
	RMSEA	<0.08	0.033	Qualified
	GFI	>0.80	0.939	Qualified
	AGFI	>0.80	0.923	Qualified
Value-added fitting index	IFI	>0.90	0.980	Qualified
	TLI	>0.90	0.976	Qualified
	CFI	>0.90	0.980	Qualified
Reduced fitting index	PGFI	>0.50	0.745	Qualified
	PNFI	>0.50	0.807	Qualified
	PCFI	>0.50	0.835	Qualified

**Table 4:** Path coefficient testing of structural equation model.

Path	Non-Standardized Path Coefficient	S.E.	C.R.	*p*	Standardization Coefficient	Result
Human–computer interaction experience → Destination image	0.891	0.09	9.889	<0.01	0.617	Valid
Human–computer interaction experience → Destination satisfaction	0.555	0.093	5.967	<0.01	0.391	Valid
Destination image → Destination satisfaction	0.35	0.057	6.147	<0.01	0.356	Valid
Human–computer interaction experience → Destination loyalty	0.457	0.101	4.535	<0.01	0.268	Valid
Destination image → Destination loyalty	0.172	0.06	2.86	0.004	0.146	Valid
Destination satisfaction → Destination loyalty	0.559	0.065	8.613	<0.01	0.465	Valid
Human–computer interaction experience → Sense immersion.	1.031	0.095	10.851	<0.01	0.720	
Human–computer interaction experience → Perceived personalization	1.141	0.101	11.277	<0.01	0.741	
Human–computer interaction experience → Perceptual controllability	1.139	0.1	11.433	<0.01	0.790	
Human–computer interaction experience → Perceived responsiveness	1				0.667	

**Table 5:** Bootstrap analysis of mediation.

Path	Effect	Estimate	S.E.	95% Confidence Interval	
				Lower	Upper
Human–computer interaction experience → destination image → destination loyalty	Indirect effect	0.153	0.076	0.003	0.293
	Direct effect	0.457	0.129	0.234	0.744
	Total effect	0.610	0.121	0.402	0.870
Human–computer interaction experience → destination satisfaction → destination loyalty.	Indirect effect	0.310	0.080	0.173	0.490
	Direct effect	0.457	0.129	0.234	0.744
	Total effect	0.768	0.137	0.538	1.069
Human–computer interaction experience → destination image → destination satisfaction → destination loyalty.	Indirect effect	0.174	0.050	0.080	0.279
	Direct effect	0.457	0.129	0.234	0.744
	Total effect	0.632	0.131	0.397	0.909

## Data Availability

The original contributions presented in this study are included in the article. Further inquiries can be directed to the corresponding author(s).
